# Comparison of MSCs and Muse cells: the possible use for healthspan optimization

**DOI:** 10.1007/s10522-025-10275-2

**Published:** 2025-07-02

**Authors:** Mari Dezawa

**Affiliations:** Department of Stem Cell Biology and Histology, Tohoku University Graduate School of Medicine, 2-1 Seiryo-Machi, Aoba-Ku, Sendai, 980-8575 Japan

**Keywords:** Intravenous injection, Sphingosine-1-P, Cell replacement, Phagocytosis, Tissue repair, Tissue rejuvenation, Immunotolerance

## Abstract

The exploration for safe, effective intervention strategies to improve longevity and aging-related diseases is attracting attention to prolong the healthy lifespan. Since aging is based on cellular changes, including telomere attrition, DNA damage, and mitochondrial dysfunction, therapies related to stem cells are expected to be a rational strategy for solving aging problems at the cellular level. Mesenchymal stem cells (MSCs) are an easily accessible, safe candidate, as they supply paracrine factors and extracellular vesicles to deliver pleiotropic effects for aging tissues. Multilineage-differentiating stress enduring (Muse) cells represent endogenous, reparative macrophage-like/pluripotent-like stem cells distributed in various tissues, including extraembryonic tissues such as the umbilical cord, and are also found in MSCs as a small percentage of the total population. Muse cell characteristics are different from those of MSCs. Intravenously injected Muse cells sharply sense the universal damage signal sphingosine-1-P and selectively migrate to damaged tissue rather than being trapped in the lung, phagocytose damaged/apoptotic cells in the tissue and directly differentiate into the same cell type. Muse cells then repair the three dimensional structure of the tissue by replacing multiple tissue component with healthy cells through pluripotent-like differentiation. Clinical trials have shown that HLA-mismatched donor Muse cells escape immune rejection and survive in the recipient tissue for an extended period without immunosuppressant treatment. Therefore, the pleiotropic bystander effects of Muse cells are more potent than those of MSCs. Due to heterogeneity, the properties of MSCs are still not fully understood; they have limited differentiation ability into osteogenic, chondrogenic, and adipogenic cells, and the main biological action in vivo is bystander effects. Muse cells are key, not only to the medical benefits of MSCs, but also to their potential use in anti-aging therapy. Enriching and purifying Muse cells will significantly enhance the therapeutic effect of MSCs, leading to further expansion of the use of MSCs. This review discusses the fundamental differences between MSCs and Muse cells and their potential applications in anti-aging therapy.

## Introduction

Aging is currently one of the most significant social problems worldwide. During aging, cells and tissues undergo functional decline, making many types of organs susceptible to aging-related diseases. The excess disease burden caused by population aging has brought enormous financial pressure to countries via increasing health care costs. Exploring safe, effective intervention strategies to prevent aging-related diseases and prolong a healthy, meaningful lifespan is a significant issue.

Cellular changes during aging are closely associated with telomere attrition, telomere deficiency, DNA damage relevant to DNA methylation and histone modification, mitochondrial dysfunction, accumulation of oxidative stress, loss of nicotinamide adenine dinucleotide levels, impaired macro-autophagy, and so on (Aguilar-Hernandez et al. 2023; Lopez-Otin et al. 2023). These lead to stem cell exhaustion, inflammation, loss of protein balance, deregulated nutrient sensing, altered intercellular communication, apoptosis, and dysbiosis (Kalamakis et al. 2019). Aging is characterized by chronic inflammation and immunosenescence. In particular, T cell aging is manifested by immunodeficiency and inflammatory responses (Pajak et al. 2023). As aging is a phenomenon based on changes at the cellular level, its effects extend to tissues throughout the body.

With aging, the skin experiences wrinkles, sagging, solar lentigo, and pigmentation, and the musculoskeletal system experiences a decrease in muscle mass (sarcopenia), decreased bone density (osteoporosis), cartilage degeneration, and joint hardening. Cardiac, hepatic, renal, digestive, and absorptive functions decline in the visceral system (Lopez-Otin et al. 2023). A decrease in basal metabolism makes it easier for fat to accumulate and may promote abnormalities in blood sugar and cholesterol levels. In the central nervous system, cognitive function declines. Particularly, short-term memory deteriorates and the ability to acquire new information becomes more difficult (Lopez-Otin et al. 2023). Aging also involves the degradation of eyesight, hearing, taste, smell, and balance, as well as an increase in the incidence of erectile in men (Lopez-Otin et al. 2023).

These age-related changes may be alleviated to some extent by careful lifestyle and nutritional approaches, such as enhanced physical activity and caloric restriction. However, proactive anti-aging treatments will be required to improve longevity more efficiently. These treatments could include interventions like cosmetic procedures or chemical/pharmacological therapies to target physical appearance and/or biological cellular aging, thus preventing or reversing the harmful effects of (Muradian 2024).

## Strategies for anti-aging using cosmetic and medical treatments

Among various target organs, anti-aging approaches for the skin are diverse. For cosmetic treatment, retinoids, hyaluronic acid, vitamin C, and growth factors are generally used for promoting cell regeneration and skin repair. For medical procedures, chemical peels for removing damaged outer skin layers, laser resurfacing that stimulates collagen production and reduces spots, and micro-needling that triggers collagen and elastin production, are common approaches (Zouboulis et al. 2019). However, these cosmetic and medical approaches are only temporary and are limited in terms of effectiveness. In that respect, biological and regenerative therapies are expected to provide a more fundamental and lasting solution.

Platelet-rich plasma therapy is used for skin and orthopedic diseases and administers a concentrated suspension of platelets derived from a patient’s blood (Everts et al. 2020; Phoebe et al. 2024). It is expected to stimulate collagen production in the skin, improve hair loss, and alleviate osteoarthritis and other orthopedic diseases.

Senolytics remove senescent cells that fall into irreversible cell cycle arrest and become resistant to apoptosis. Notably, senescent cells adopt a senescence-associated pro-inflammatory secretory phenotype (SASP) that produces cytokines, chemokines, proteases, and other factors to promote inflammation and cellular damage around the senescent cells, leading to the aging of surrounding tissues (Guarente et al. 2024). Drugs such as Dasatinib, quercetin, and Navitoclax are attracting attention as one of the fundamental treatments for aging because they selectively induce the death of senescent cells by targeting specific signaling pathways and survival mechanisms that differ from those in healthy cells (Guarente et al. 2024). However, careful consideration is required to optimize the therapeutic benefits and minimize potential risks, since different cell types and tissues respond differently (Tombak et al. 2025).

Besides these therapies, another approach that may provide a fundamental solution to the treatment of age-related physiological decline, is stem cell-based therapies (Muradian 2024).

## Anti-aging therapies related to stem cells

### Conditioned medium

Conditioned medium, namely, the culture medium of stem cells, contains various factors relevant to tissue protection and regeneration. Among stem cells, umbilical cord (UC)- and adipose tissue-derived mesenchymal stem cells (MSCs) are the most frequently used stem cells in cosmetics and medical treatment. The conditioned medium contains epidermal growth factor (EGF), basic fibroblast growth factor (bFGF), and transforming growth factor-beta (TGF-β), which are essential in cell growth and, particularly, in skin tissue maintenance. It also contains vascular endothelial growth factor (VEGF), platelet-derived growth factor (PDGF), hepatocyte growth factor (HGF), keratinocyte growth factor (KGF), and insulin-like growth factor (IGF) that accelerate tissue regeneration (Alquraisy et al. 2024). Another advantage of conditioned medium is its fewer stringent regulatory constraints compared to that of stem cell therapy.

### Extracellular vesicles (EVs)

EVs contain a variety of heterogeneous nanovesicles secreted by almost all cell types, primarily for intercellular communication and maintaining cellular homeostasis. EVs have been broadly categorized into several subtypes based on their size and origin, such as exosomes (from ~ 30 to less than 200 nm), microvesicles (100 ~ 1000 nm), and apoptotic bodies (0.5 ~ 2 microns) (Rather et al. 2023; Rudnitsky et al. 2024, 2025). In addition, new subtypes of EVs are continually being discovered (Rather et al. 2023). All cell types secrete EVs, and those secreted by stem cells, such as MSCs, provide cell-free nanotherapeutics (Gui et al. 2024; Rudnitsky et al. 2025). Since they are known to contain metabolites, proteins, various nucleic acids including microRNAs and noncoding RNAs, and even organelles such as mitochondria, they can function as biomolecule cargos and are shown to have tissue protection, anti-inflammation, anti-oxidative stress, and wound healing-promoting abilities (Rather et al. 2023; Rudnitsky et al. 2025). Among MSCs, human UC-MSC-derived EVs are derived from the youngest adult stem cell source and are thus expected to have significant potential applications in anti-aging therapy (Zhang et al. 2024).

### Stem cells

Compared to conditioned medium and EVs, stem cell therapy could be a more direct approach for controlling aging because cells can produce the efficient factors in a more powerfull and stable manner. Among various stem cells that are potential candidates for anti-aging treatment, MSCs are very attractive from the practical point of view due to their ease of access, preparation, and safety, as they are non-tumorigenic. They secrete components such as paracrine factors and EVs that deliver a pleiotropic bystander effects, synergistically supporting tissue reconstruction and repair (El Assaad et al. 2024; Rudnitsky et al. 2025; Xu & Song 2025; Zarei & Abbaszadeh 2019).

Another candidate is Multilineage-differentiating stress enduring (Muse) cells. Muse cells are endogenous, reparative macrophage-like pluripotent-like stem cells distributed in various tissues and organs in our body as well as extraembryonic tissues, such as the UC. They have already been applied to clinical trials and have been reported to be safe and therapeutically effective in heart, brain, and skin diseases (Fujita et al. 2021b; Koda et al. 2024; Niizuma et al. 2023; Noda et al. 2020; Sato et al. 2024; Yamashita et al. 2023).

The following sections focus on MSCs and Muse cells and their use for anti-aging therapy.

## MSCs

MSCs are defined as cells positive for mesenchymal markers CD29, CD73, CD90, and CD105, and are negative for CD45, CD31, CD34, and CD117, obtained from various mesenchymal tissues such as the bone marrow (BM), UC, and adipose tissue as adherent cells (Table 1) (Dominici et al. 2006). They can be collected from accessible sources and are expandable to a clinical scale. However, they are a crude, heterogeneous cell population (Dominici et al. 2006).

**Table 1:**
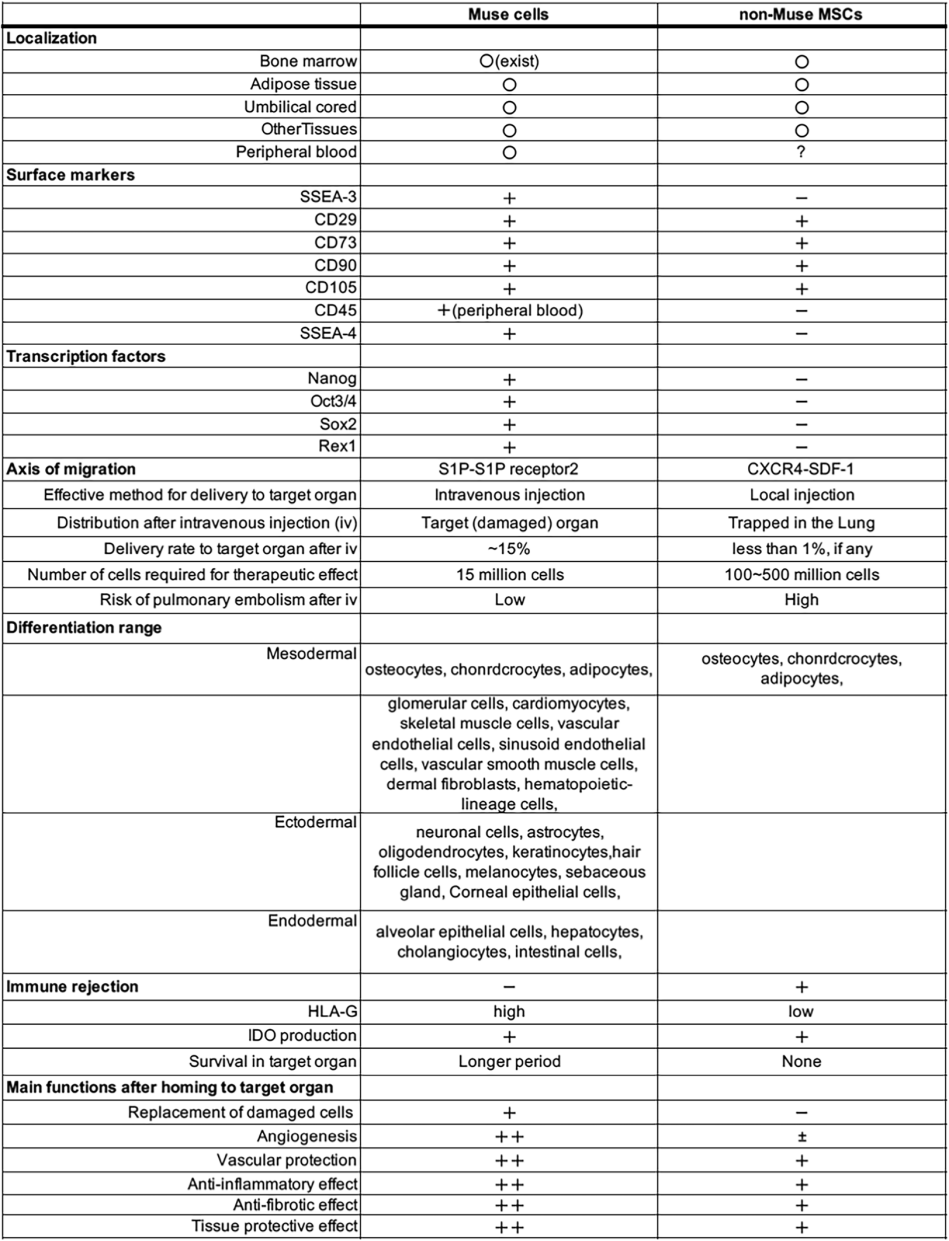
Comparison between Muse cells and non-Muse MSCs

Localization, (Yamashita et al. 2021) surface marker expression, (Kuroda et al. 2010; Wakao et al. 2011) the expression of transcription factors, (Kuroda et al. 2010; Wakao et al. 2011) migration axis, (Yamada et al. 2018; Yamashita et al. 2021) differentiation ability, (Fujita et al. 2021; Guo et al. 2020; Hosoyama et al. 2018; Katagiri et al. 2016; Kuroda et al. 2010; Kushida et al. 2024; Uchida et al. 2017b; Wakao et al. 2011, 2022; Win et al. 2024; Yamada et al. 2018) immune rejection, (Kuroda et al. 2022) and main functions in the homed tissue (Minatoguchi et al. 2024) are compared between Muse cells and non-Muse MSCs

The main action of MSCs when infused in vivo is the pleiotropic bystander effect, in which tissues are protected through the production of cytokines relevant to endogenous tissue stem cell/progenitor activation and tissue regeneration [such as HGF, IGF, PDGF, EGF, brain-derived neurotrophic factor (BDNF), and nerve growth factor (NGF)], immune modulation [such as Interleukin (IL)-6, IL-10, IL-1Ra, TGF-β, tumor Necrosis Factor-stimulated Gene-6 (TSG-6), prostaglandin E2 (PGE2), indoleamine 2,3-dioxygenase (IDO)], and vascular protection and neovascularization (such as VEGF and bFGF), that synergistically contribute to creating a microenvironment supportive of tissue repair (Kuroda et al. 2022; Rudnitsky et al. 2025; Zarei & Abbaszadeh 2019).

MSCs have been shown to differentiate into osteogenic, chondrogenic, and adipogenic cells, but wide-ranging pluripotent-like differentiation across the oligo-lineage boundaries between mesodermal-ectodermal and mesodermal-endodermal rarely occurs (Table 1). Thus, their pluripotency has long been a topic of debate. MSCs and MSC-derived EVs have been used in clinical trials for many diseases and for commercial anti-aging interventions (Rudnitsky et al. 2024, 2025). In both cases, the main effect is due to a bystander effect, provided for a specific period after administration. Their efficacy is relatively independent of the donor age in humans but not in rodents (Rudnitsky et al. 2025).

More detailed information on the application of MSCs in anti-aging treatments can be found elsewhere (Rudnitsky et al. 2024, 2025).

## Muse cells

Muse cells are endogenous reparative stem cells found as pluripotent surface marker stage-specific embryonic antigen (SSEA)-3-positive in the BM (~ 0.01% ~ 0.03% of the mononuclear fraction), peripheral blood (~ 0.01% ~ 0.2% of the mononuclear fraction), the connective tissue of every organ, and the extraembryonic tissues such as the UC and amnion (Dezawa 2025; Kuroda et al. 2010; Kushida et al. 2024; Leng et al. 2019; Ogawa et al. 2022; Ogura et al. 2014). They are also found in cultured MSCs and fibroblasts from one to several percent of the cell population (Kuroda et al. 2010; Wakao et al. 2011).

Muse cells have dual aspects of monocytes/macrophages and pluripotent stem cells (Wakao et al. 2022). Furthermore, as shown in clinical trials, HLA-mismatched allogeneic Muse cells can be directly administered to patients without immunosuppressants and remain integrated in the recipient tissue for an extended period due to a specific immunotolerance mechanism (Dezawa 2025; Fujita et al. 2021b; Koda et al. 2024; Kuroda et al. 2022; Minatoguchi et al. 2024; Niizuma et al. 2023; Noda et al. 2020; Sato et al. 2024; Yamashita et al. 2023).

**Fig. 1:**
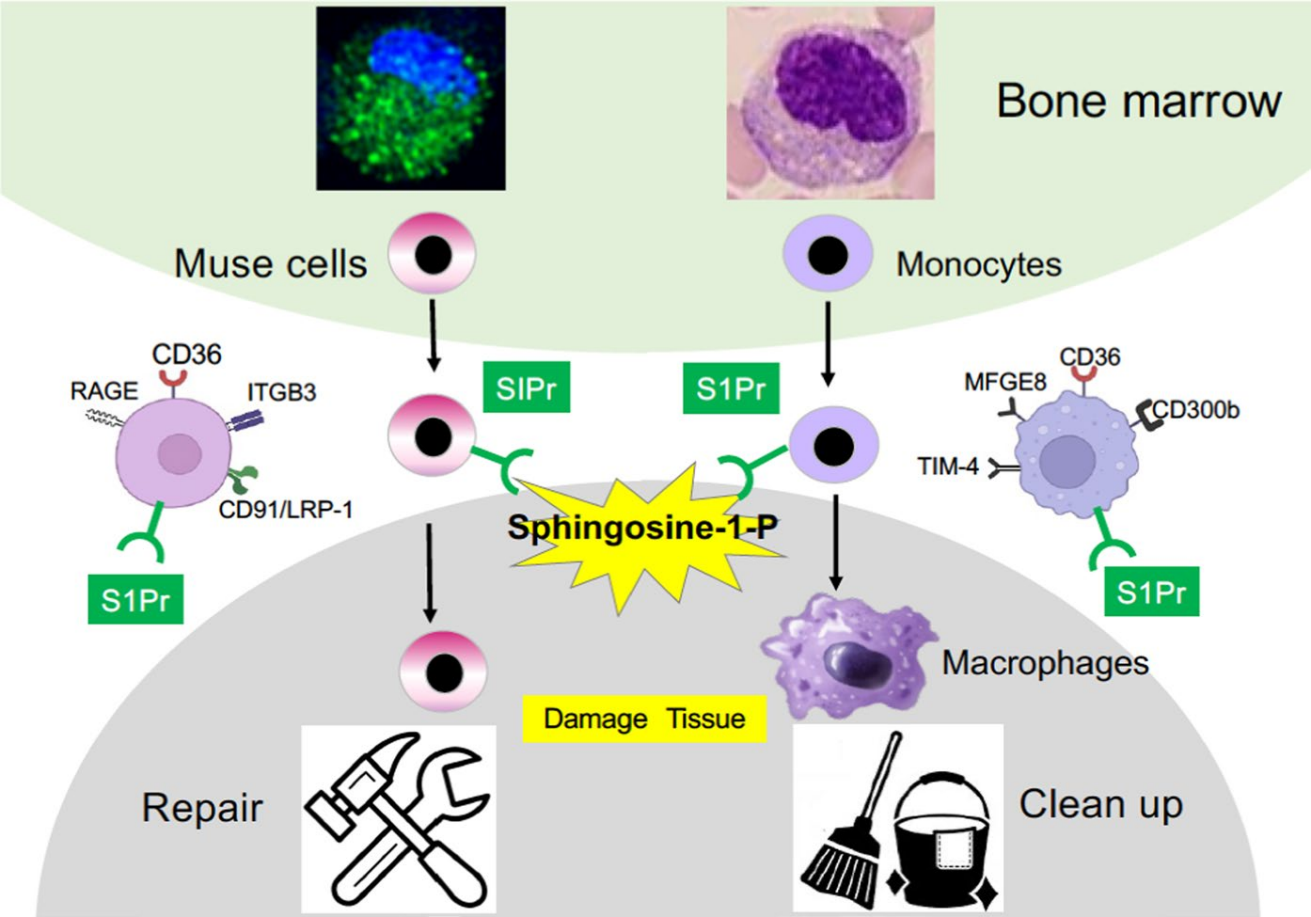
Similarity between Muse cells and monocytes/macrophages (Wakao et al. 2022). Both are similar in the reserve site (the BM), morphology of nucleus, size, recruitment to the damaged site by sensing sphingosine-1-P (S1P), expression of phagocytosis receptors, and phagocytic activity. Monocytes/macrophages are professional phagocytes for cleaning the tissue, but Muse cells repair the tissue by cell replacement through phagocytosis-dependent differentiation. This figure was reproduced and modified with permission (Dezawa 2025).

Muse cells are similar to monocytes/macrophages in size (10–15 µm in diameter), morphology of the nucleus (bean-shaped or heart-shaped), sensing sphingosine-1-P (S1P) to be recruited to damaged tissue, and the expression of receptors for phagocytosis (Fig. 1) (Sato et al. 2020; Wakao et al. 2022; Yamada et al. 2018). They express monocyte/macrophage markers such as CD45 (in peripheral blood-Muse cells), CD80, interleukin-10 (IL10), and toll-like receptor 2 (TLR2) (Wakao et al. 2022).

**Fig. 2:**
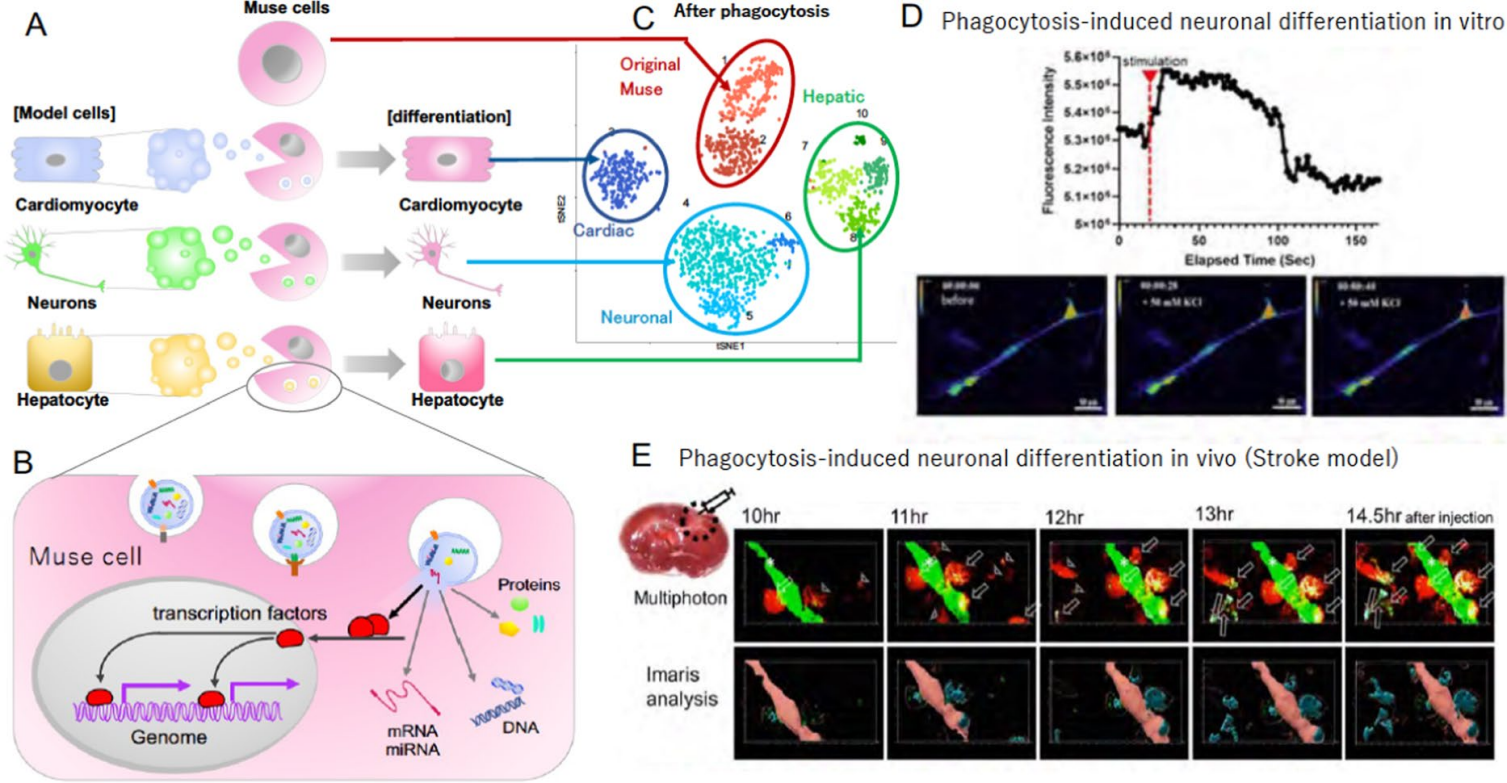
Phagocytosis-dependent differentiation mechanism in Muse cells (Wakao et al. 2022). **A** Pluripotent Muse cells can differentiate into the same cell type as the phagocytosed apoptotic cells. **B** The mechanism of how Muse cells recycle signals from the up-taken damaged/apoptotic cells, such as transcription factors, necessary for differentiation. **C** Single-cell RNA sequencing of human Muse cells after phagocytosing apoptotic cell fragments of mouse hepatic (Hepatic), mouse cardiac (Cardiac), and rat neural (Neuronal) cells at 7 days. Human Muse cells before phagocytosis is “Original Muse”. **D** Intracellular calcium dynamics in green fluorescent protein (GFP)-based Ca calmodulin probe (GCaMP) after biochemical depolarization with 50 mM KCl in human Muse cell-derived neuronal cells induced by phagocytosis. **E** Neuronal differentiation of mCherry-human Muse cells introduced with NEUROD1-promoter-CFP in C57BL/6-Tg (CAG-EGFP) mouse focal stroke model. mCherry-human Muse cells phagocytosed GFP( +) recipient neural apoptotic fragments. They became NeuroD1-CFP positive in the mouse focal brain ischemia region, shown by TTC staining (upper left corner of (E). The figures were reproduced with permission (Wakao et al. 2022).

On the other hand, they are different from monocytes/macrophages in the expression of pluripotency markers such as Oct3/4, Nanog, Sox2, and Rex1, differentiation into endodermal-, mesodermal-, and ectodermal-lineage cells at a single cell level, and self-renewal ability (Table 1) (Kuroda et al. 2010). Their differentiation direction is controlled by what they phagocytose both in vitro and in vivo (Fig. 2A): they recycle signals necessary for differentiation, such as transcription factors, from the up-taken damaged/apoptotic cells, and quickly differentiate into the same cell type on a daily basis, as demonstrated by single-cell RNA sequencing (Fig. 2B, C) (Wakao et al. 2022). Fusion with mature cells was not considered the primary mechanism of Muse cell differentiation, as shown by the live image of phagocytosis-dependent differentiation in vitro and in vivo, as well as by fluorescence in situ hybridization (FISH) data (Iseki et al. 2017; Uchida et al. 2017b; Wakao et al. 2022; Yamada et al. 2018).

This differentiation mechanism sharply contrasts not only monocytes/macrophages, but also other stem cells. In embryonic stem (ES) cells and induced pluripotent stem (iPS) cells with very low phagocytosis activity (Wakao et al. 2022), cytokine and/or gene introduction-dependent differentiation induction is the main strategy for controlling differentiation, which generally requires several weeks to months with multiple steps. In a damaged tissue microenvironment, various positive and negative factors for stem cell survival and differentiation are mixed and do not sequentially supply the cytokine cocktail according to the differentiation step, as used in the in vitro differentiation induction of ES and iPS cells. The phagocytosis-dependent differentiation adopted by Muse cells is, therefore, a rational system suited to the reparative system of the living body, which enables replacing damaged/apoptotic cells with healthy cells in a short time period.

**Fig. 3:**
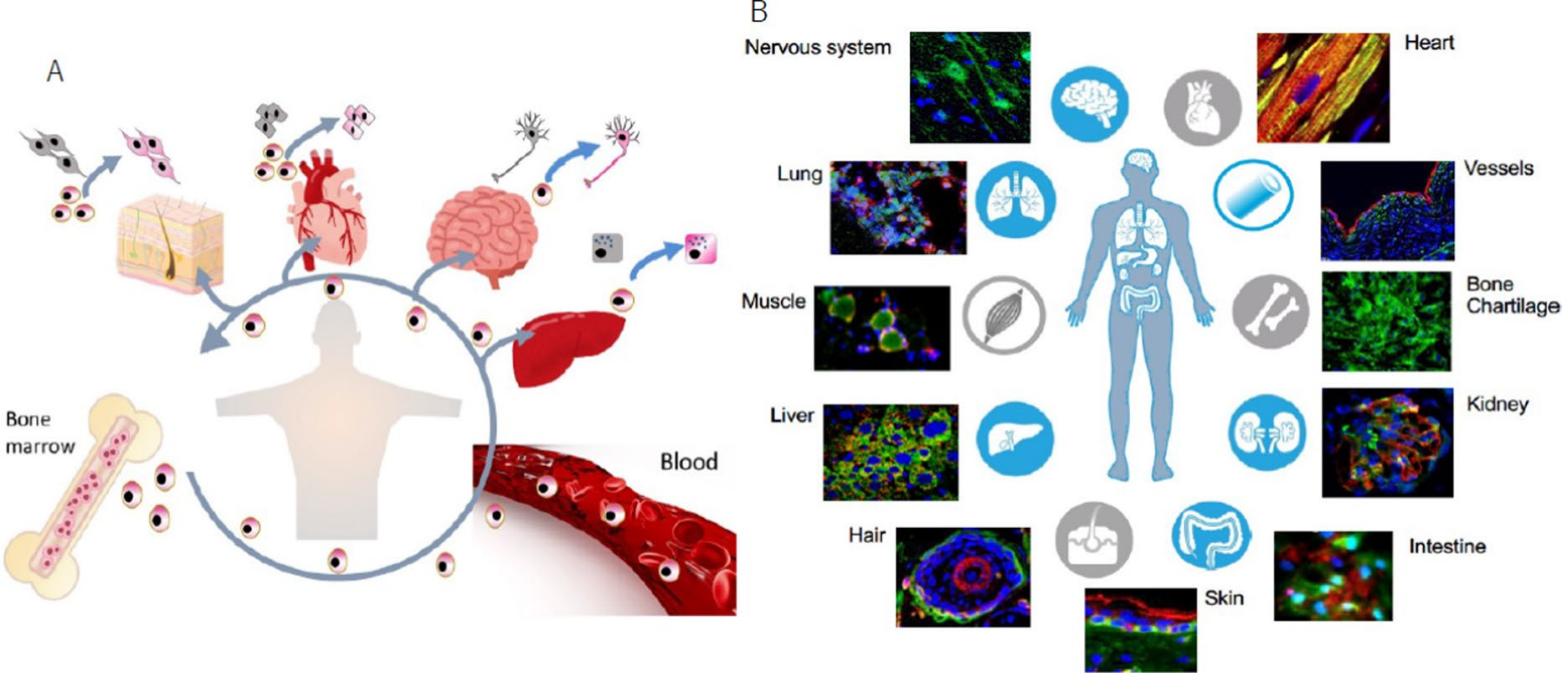
The dynamics of endogenous Muse cells and their reparative activity. **A** Reparative activity of endogenous Muse cells. Muse cells in the BM are constantly mobilized to the peripheral blood and supplied to every organ, where they replace minutely damaged/apoptotic by phagocytosis-dependent differentiation. The figures were reproduced with permission (Yamashita et al. 2021). **B** Pluripotent Muse cells (GFP +) can differentiate into various cell kinds.

Endogenous Muse cells have been suggested to be correlated with an individual’s ability to repair damaged or aging tissue. The main reserve of Muse cells is in the BM. They are constantly mobilized into the peripheral blood and distributed to organs throughout the body by the bloodstream (Fig. 3A) (Kuroda et al. 2010; Sato et al. 2020; Yamashita et al. 2021). They are also thought to be involved in tissue homeostasis by replacing damaged cells through phagocytosis and differentiation in each location (Fig. 3B). In a rabbit myocardial infarction model, endogenous Muse cells were mobilized from the BM through the bloodstream to the infarcted area and spontaneously differentiated into cardiomyocytes in the ischemic heart tissue (Minatoguchi et al. 2025). Clinical data has also showed that in patients with acute myocardial infarction or who have undergone liver surgery, early functional recovery and avoiding transition to chronic disease were achieved when endogenous Muse cells were successfully mobilized into the peripheral blood during the acute phase (Kikuchi et al. 2022; Tanaka et al. 2018). These findings suggest that the ratio and activity of endogenous Muse cells are related to an individual’s reparative activity.

## The fundamental difference between non-Muse MSCs and Muse cells

As a subpopulation of MSCs, corresponding to 1 ~ several percent of total cells, Muse cells express mesenchymal markers CD29, CD73, CD90, and CD105, and are negative for CD31, CD34, and CD117 (Wakao et al. 2011). Peripheral blood-Muse cells are positive for CD45, a white blood cell marker, while MSCs are consistently negative for CD45 (Sato et al. 2020). Like MSCs, Muse cells are non-tumorigenic and produce various factors with anti-inflammatory, anti-fibrotic, and tissue-protective properties (see 11. Tissue protection, anti-inflammation, immunomodulation, and anti-fibrosis**)** (Kuroda et al. 2010, 2022; Li et al. 2024).

However, when MSCs are separated into SSEA-3( +) Muse cells and SSEA-3(-) non-Muse MSCs (cells other than MSCs, corresponding to ~ 97–98% of the total MSCs), fundamental differences can be seen between them. One of them is in their ability to differentiate, as mentioned above (Table 1) (Ogura et al. 2014; Wakao et al. 2011).

**Fig. 4:**
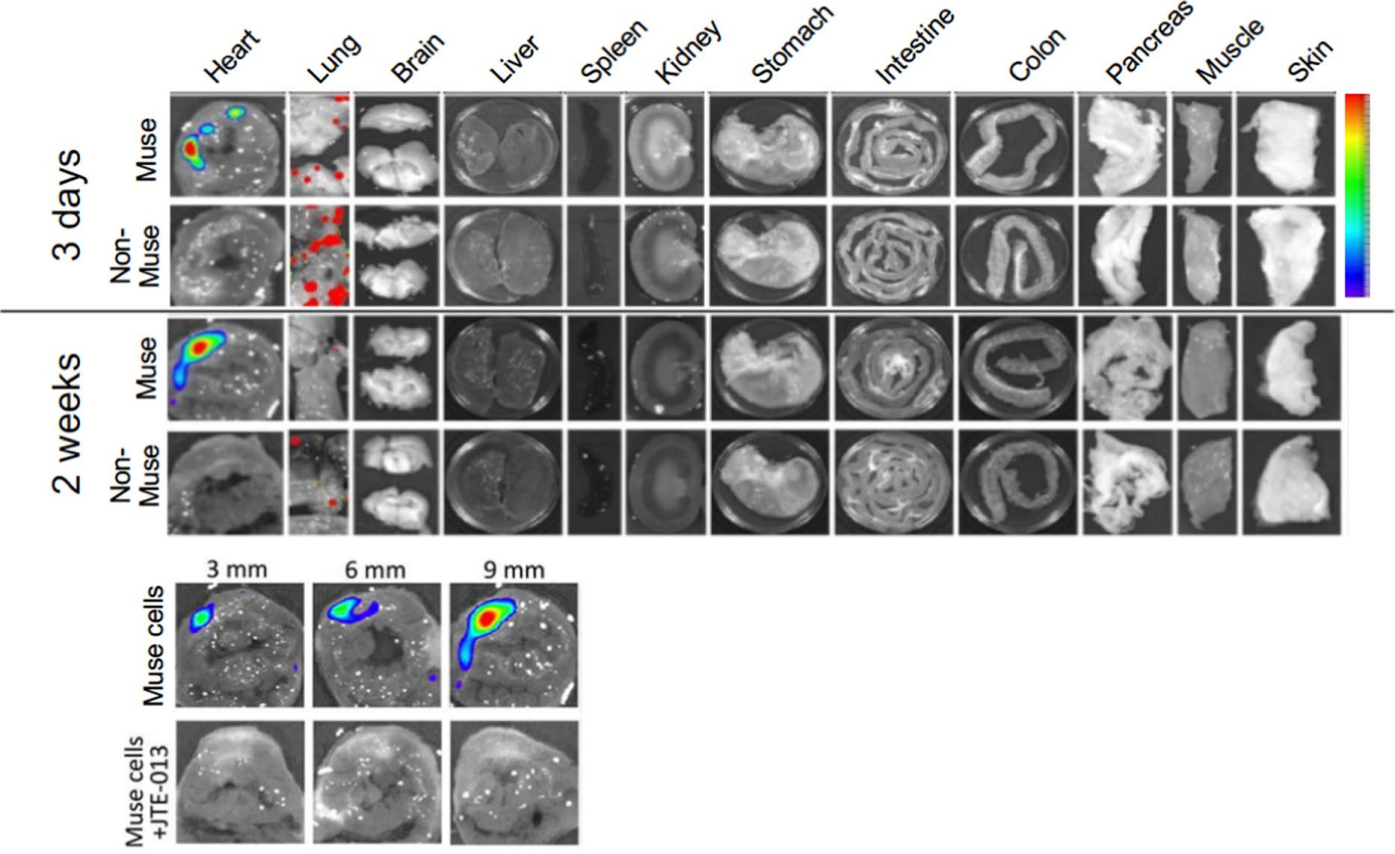
Migration ability of Muse cells and non-Muse MSCs to damaged site (Yamada et al. 2018). Rabbit acute myocardial infarction model that received Nano-lantern labeled human BM-Muse cell and -non-Muse MSC IV. **A** Muse cells migrated and homed into the ischemic heart region at day 3. Non-Muse MSCs were under the detection limit in the heart and were distributed in the lung. At 2 weeks, Muse cells still remained in the heart, while non-Muse MSCs disappeared from the body. **B** Engraftment of allograft Muse cells with and without the S1PR2 antagonist JTE-013 at 2 weeks. Migration and homing of Muse cells were almost abolished by the presence of JTE-013. The figures were reproduced with permission (Yamada et al. 2018).

Intravenously injected Muse cells pass through the lung capillary, selectively migrate to and accumulate in damaged tissue by sensing S1P, which is produced by damaged cells through the conversion of sphingosine into S1P in the cell membrane (Table 1, Fig. 4). They then differentiate into the damaged/apoptotic cell type through a phagocytosis-dependent mechanism, and replace damaged/apoptotic cells with healthy functional cells to aid in tissue repair (Wakao et al. 2022; Yamada et al. 2018). The S1P-S1P receptor as the central axis of Muse cell migration was supported by the fact that S1P receptor suppression by the antagonist or by siRNA introduction in Muse cells abolished the specific migration of Muse cells to damaged tissue (Fig. 4) (Yamada et al. 2018). On the contrary, the migration of non-Muse MSCs is mainly controlled by CXCR4-SDF-1, irrelevant to damage signals, and thus, they are trapped in the lung and do not reach the damaged tissue (Table 1) (Fujita et al. 2021; Shono et al. 2021; Yamada et al. 2018; Yang et al. 2015). Muse cells remain integrated in the recipient organ as differentiated cells long term by escaping immune rejection. Contrarily, non-Muse MSCs disappear from the body within several weeks (Fig. 4) (Yamada et al. 2018). Although the detailed mechanism for escaping immune rejection in Muse cells is still not fully understood, one suggested mechanism is that Muse cells express HLA-G, relevant to immunotolerance in the placenta, at a higher percentage than MSCs (Table 1) (Kuroda et al. 2022; Yamada et al. 2018).

Overall, Muse cells can structurally and functionally repair various kinds of damaged tissues, but the main action of non-Muse MSCs in vivo is a transitory tissue protection effect.

Notably, ~ 15% of Muse cells are homed to a damaged site, while fewer than 1% of MSCs or non-Muse cells, if any, are homed to the damaged site, after intravenous injection (IV) (Yamada et al. 2018). Due to their high integration efficiency into damaged organs, 15 million Muse cells were injected intravenously per patient, 10–30 times less than the cell numbers used in MSC treatments to deliver higher therapeutic effects (Table 1) (Minatoguchi et al. 2024).

## Resistance against stresses, including genotoxic stress, is higher in Muse cells than in MSCs/non-Muse MSCs

Muse cells showed higher stress tolerance and survivability than MSCs/non-Muse MSCs in nutrient-deprived trypsin solution for at least 16 h, which is partly explained by the higher expression of factors related to stress tolerance, such as 14–3-3 proteins and serpins (Alessio et al. 2017). Muse cells also showed higher resistance against genotoxic stress than in MSCs and non-Muse MSCs: after ultraviolet light and H_2_O_2_ exposure, Muse cells exhibited lower rates of apoptosis and senescence than MSCs and non-Muse MSCs due to higher expression of ataxia-telangiectasia mutated kinase and γ-H2AX, relevant to DNA repair, and of enzymes related to non-homologous end-joining (Alessio et al. 2018). Repair of damaged DNA was completed within 6 h in Muse cells, whereas it took ~ 48 h in MSCs and non-Muse MSCs (Alessio et al. 2018). A higher capacity for stress tolerance and DNA repair contributes to the low risk of tumorigenesis and resistance to the accumulation of mutations in Muse cells, suggesting their suitability for anti-aging treatments.

## Comparative therapeutic effects of Muse cells and MSCs/non-Muse MSCs in repairing age-related organ damage

**Fig. 5:**
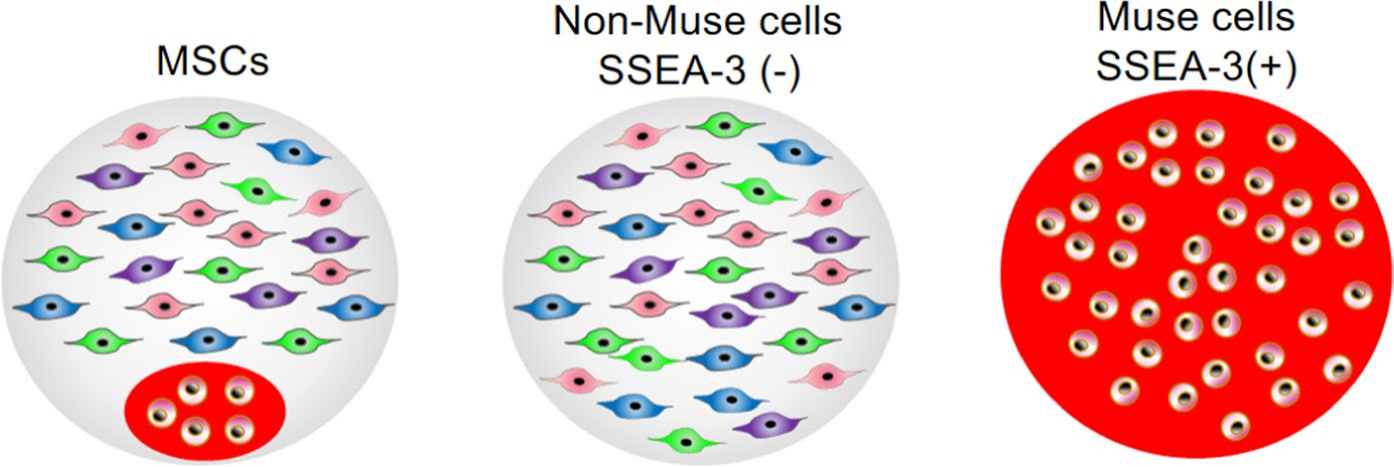
Schematic diagram of MSCs, non-Muse MSCs, and Muse cells. MSCs contain 1 ~ several percent of SSEA-3( +) Muse cells. SSEA-3(-) non-Muse MSCs are cells other than Muse cells in MSCs. In the most of preclinical studies, the same number of MSCs, non-Muse MSCs, and Muse cells were administered to animal models.

In all the preclinical studies to date, Muse cells and non-Muse cells, collected as SSEA-3( +) and SSEA-3(−) cells from BM-, adipose-, dermal- and UC-MSCs, respectively, and MSCs that contain several percent of Muse cells, were directly administered intravenously without in vitro differentiation induction, unlike ES and iPS cells. Unless otherwise stated, the same number of cells were administered intravenously in the Muse cell, non-Muse MSC, and MSC groups (Fig. 5). All the clinical trials were conducted by IV of HLA-mismatched donor Muse cells (15 million cells per single dose) without immunosuppressant treatments.

### Skin and hair

**Fig. 6:**
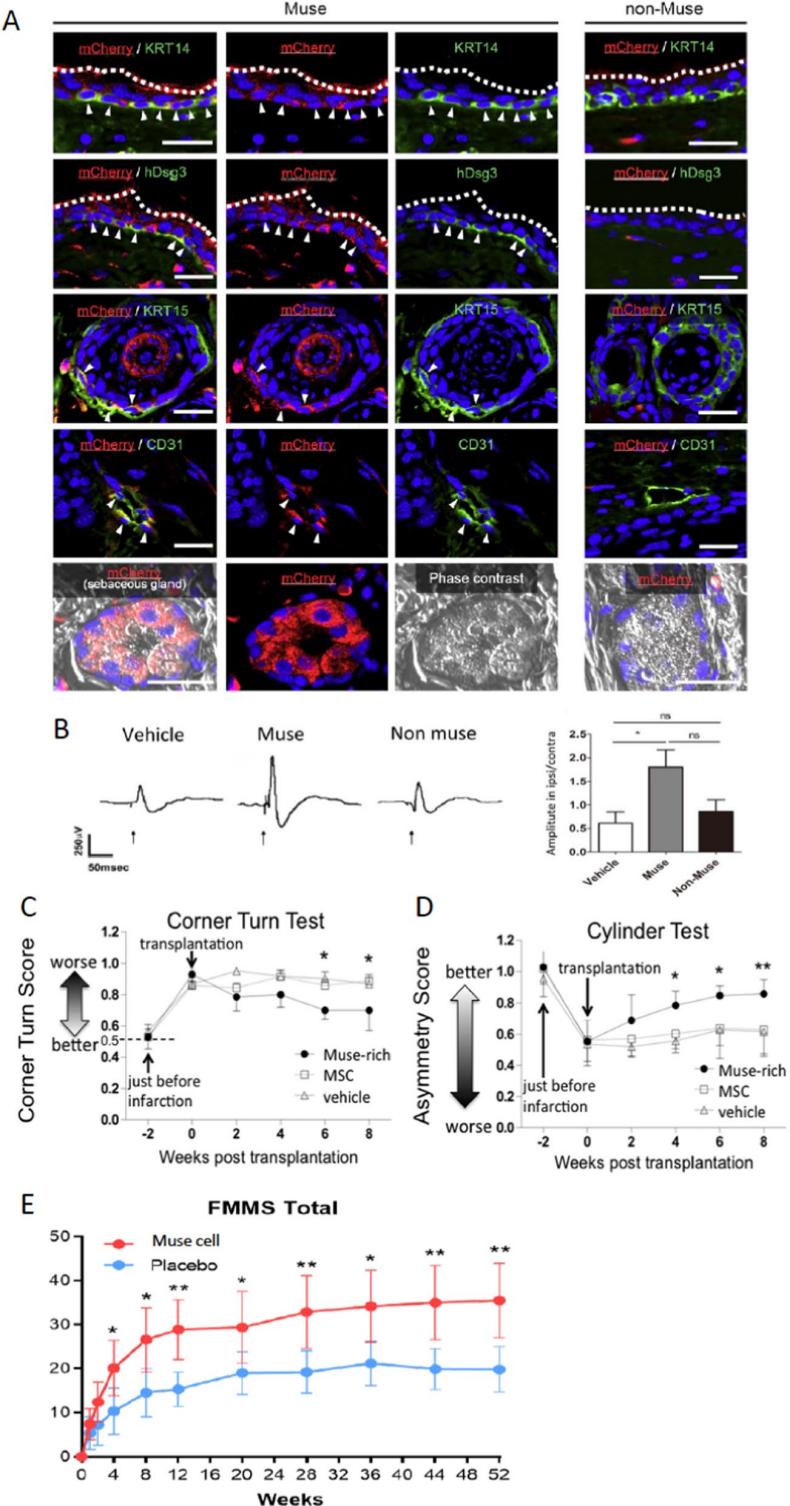
Comparison of therapeutic effects between Muse cells, non-Muse MSCs and MSCs (Part 1). **A** Collagen17-/- epidermolysis bullosa mouse model that received intravenous injection of mCherry-labeled human BM-Muse cells and -non-Muse MSCs (4 weeks). mCherry-Muse cells differentiated into keratinocytes, hair follicular cells, vascular cells, and sebaceous gland, but mCherry-non-Muse MSCs did not integrate into the skin and thus their differentiation was not observed. The figures were reproduced with permission (Fujita et al. 2021). **B** Rat middle cerebral artery occlusion model that received human dermal-Muse cell and non-Muse MSCs IV (84 days after). Representative somatosensory evoked potentials (SEP) in the vehicle, Muse, and non-Muse MSC groups. The graph shows the SEP amplitude on ipsilateral side to that on contralateral side. Significant difference was recognized between the vehicle and Muse groups. *: p < 0.05. The figures were reproduced with permission (Uchida et al. 2016). **C**, **D** Mouse subacute lacunar stroke model that received human BM-Muse cell and MSC IV. The functional recovery of the Muse group in the corner turn (C) and cylinder (D) tests was superior to the MSC group with statistical significance. *: p < 0.05. **: p < 0.01. The figures were reproduced with permission (Uchida et al. 2017a). **E** A double-blind, placebo-controlled clinical trial for subacute ischemic stroke. Adjusted mean (95% CI) change from baseline in Fugl–Meyer Motor Scale (FMMS) for both the upper and lower limbs in the Muse cell-treated and placebo-treated groups.**P < .01, ***P < .001. The figures were reproduced with permission (Niizuma et al. 2023).

Intravenously injected human BM-Muse cells selectively recruited to the blister and ulcer areas, differentiated into keratinocytes, hair follicle cells, sebaceous gland, and vascular cells in the collagen17-/- epidermolysis bullosa mouse model at 4 weeks (Fujita et al. 2021). The human-Muse cells that were integrated into the basal layer of the mouse epidermis differentiated into keratin 14 ( +) and human desmoglein 3 ( +) cells and produced human collagen-7 and − 17 at 6 months without immunosuppressant treatment, suggesting the maintenance of functionality of xenogenic Muse cells in vivo for an extended period (Fujita et al. 2021). Few intravenously injected non-Muse cells integrated into the skin or differentiated into skin components in vivo at 4 weeks (Fig. 6A) (Fujita et al. 2021).

In the streptozotocin-induced diabetes SCID mouse skin ulcer model, subcutaneously injected human adipose-Muse cells significantly accelerated wound healing compared to the human adipose-MSC treated group and even non-diabetic normal SCID mice (Kinoshita et al. 2015).

As human dermal Muse cells differentiated into fibroblasts by TGF-beta and ascorbic acid in vitro, Muse cell treatment may refill fresh juvenile fibroblasts and their products, including collagen and other extracellular matrix, into the aged skin (Yamauchi et al. 2017a).

**Clinical trials:** The safety and efficacy of donor Muse cell IV in treating epidermolysis bullosa was reported (Fujita et al. 2021b). In a phase I/II open-label, non-randomized trial, five adult dystrophic epidermolysis bullosa patients (1 man and 4 women, 26.8±12.8 years old) with 13 refractory/recurrent ulcers received a single dose of donor Muse cell IV. Two patients showed a >50% reduction in the area of the selected ulcer at 4 weeks, and overall, the combined size of the selected ulcers was significantly reduced during the 52-week observation period, with reduced pain in the ulcer area and improved serum liver enzymes. Prominent adverse events associated with the treatment were not reported, except of one patient experienced transient paresthesia (Fujita et al. 2021b).

In a double-blind, placebo-controlled clinical trial of ischemic stroke, ~24% of patients experienced a change in hair color and hair growth between 12 weeks and 52 weeks, while no patients in the placebo group experienced such changes, suggesting the efficiency of Muse cells for hair growth and color change (Niizuma et al. 2023).

### Musculoskeletal system

Intra-articular injection of human BM-Muse cells (50,000 cells) repaired the osteochondral defect in the patellar groove of immunodeficient rats. The white repaired tissue had a mostly smooth, homogenous surface in the cartilage at 12 weeks after administration in the Muse cell-treated group, while no repair tissue was detected in the non-Muse MSC (50,000 cells) group (Mahmoud et al. 2017). In the cardiotoxin-induced mouse muscle degeneration model, intravenously injected human BM-Muse cells homed into the muscle tissue, differentiated into Pax7( +) muscle stem cells, and human dystrophin( +) muscle fibers at 2 weeks (Kuroda et al. 2010).

### Central nervous system

Human BM-, adipose-, and dermal-Muse cells differentiated into NeuN( +) and MAP2( +) neurons, GST-pi( +) oligodendrocytes, and glial fibrillary acidic protein (GFAP)( +) astrocytes, but non-Muse MSCs from those sources did not, both in vitro and in vivo (Abe et al. 2020; Kajitani et al. 2021; Ogura et al. 2014; Shimamura et al. 2017; Suzuki et al. 2021; Uchida et al. 2016, 2017a; Wakao et al. 2011; Yamashita et al. 2020). Muse cells were shown to differentiate into neuronal cells after phagocytosing apoptotic neuronal cell fragments: they newly expressed Math1, NeuN, neurofilament, and Tuj1, and functional markers such as voltage-gated potassium and sodium channels, postsynaptic density protein 95, and synaptophysin from day 3 ~ 21 both in vivo (stroke model) and in vitro. Those differentiated cells exhibited a calcium-influx response to biochemical depolarization, suggesting physiological activity of differentiated Muse cells (Fig. 2D) (Wakao et al. 2022). In the mouse stroke model, human BM-Muse cells were shown to express NeuroD ~ 14.5 h after phagocytosing recipient apoptotic neuronal cell fragments in the infarct tissue (Fig. 2E) (Wakao et al. 2022). In rat stroke model, human BM-Muse cell-derived neurites were incorporated into the pyramidal tract, making synaptic connections with spinal cord neurons (Uchida et al. 2016, 2017a). Muse cells integrated into the sensory cortex formed synapses with host neurons, leading to the somatosensory evoked potential recovery (Fig. 6B) (Uchida et al. 2016). Regardless of models, statistically meaningful functional recovery was confirmed in the Muse cell-treated group in rotarod, modified neurologic severity score, BBB motor scale, corner turn, cylinder, hanging-wire, and water maze tests compared to the MSCs and/or non-Muse MSC-treated groups (Fig. 6C, D) (Shimamura et al. 2017; Suzuki et al. 2021; Uchida et al. 2016, 2017a; Yamashita et al. 2020).

**Clinical trials:** A double-blind, placebo-controlled randomized clinical trial for subacute ischemic stroke enrolled patients who were bedridden and incontinent, or needed assistance for walking, eating, and toileting, categorized as modified Rankin scale (mRS) 5 and 4, respectively (Niizuma et al. 2023). Patients received a single dose IV of donor-Muse cells or placebo without immunosuppressant 14–28 days after onset. Statistically meaningful improvements in the Fugl-Meyer Motor Scale upper limb and total scores were observed as early as 4 weeks and continued to 52 weeks (Fig. 6E). Early improvements in upper limb function, such as buttoning a shirt, signature, and eating, are generally difficult to achieve and the remarkable improvement in the upper limb function in the active group directly related to improvement of the mRS from 4~5 to 0 or 1.

A prospective, multicenter, nonrandomized, nonblinded, single-arm study was conducted on cervical spinal cord injury. Patients with C4 to C7 injury that showed modified Frankel classification B1 and B2 (average age of 49.3 ± 21.2 years) received a single dose of donor Muse cells 3 weeks after injury. The ISNCSCI motor score, the activity of daily living, and quality of life scores after treatment showed statistically significant improvements compared to those before administration (Koda et al. 2024).

Since ALS takes a chronic course, donor-Muse cells were administered multiple times (once a month for 6 months without immunosuppressants) in an open-label, non-randomized, single-arm, non-controlled clinical trial. Patients with sporadic ALS with the limb-onset clinical form were enrolled. Despite repeated administration of donor Muse cells without HLA-matching tests or immunosuppressants, the treatment was highly tolerated, without any serious adverse effects. In approximately 80% of patients, scores on the Revised Amyotrophic Lateral Sclerosis Functional Rating Scale (ALSFRS-R) remained stable for up to 10 months after initiating cell administration, whereas about 20% showed a trend toward deterioration—findings that strongly support the therapeutic potential of donor Muse cell treatment mitigating ALS symptom progression (Yamashita et al. 2023).

### Cardiovascular system

**Fig. 7:**
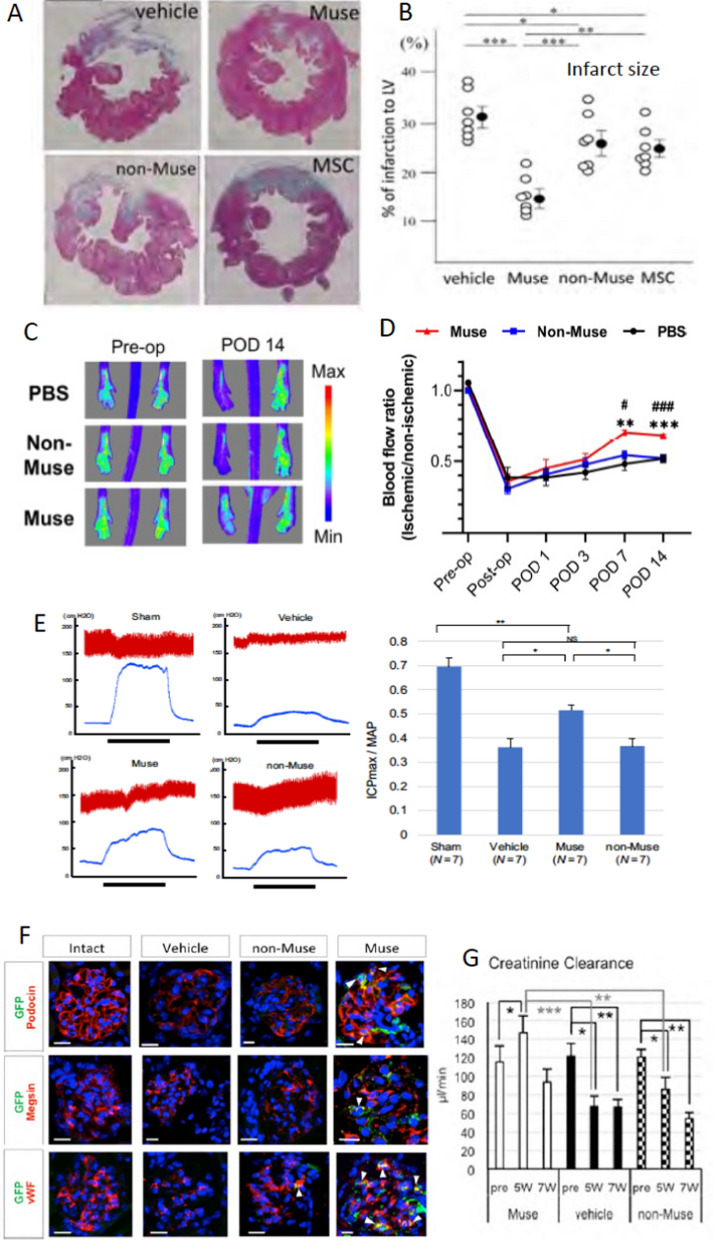
Comparison of therapeutic effects between Muse cells, non-Muse MSCs, and MSCs (Part 2). (**A**, **B)** Rabbit acute myocardial infarction model that received IV of autologous BM-Muse cells, MSCs, and non-Muse MSCs (2 months). **A** Masson trichrome staining and **B** infarct size. The Muse group showed a statistically significant reduction of the infarct size compared to the non-Muse MSC (p < 0.001) and MSC (p < 0.01) groups, with statistical significance. At the same time, there was no significance between the non-Muse MSC and MSC groups. The figures were reproduced with permission (Yamada et al. 2018). (**C**, **D**) Mouse hind limb ischemic model that received human BM-Muse cell and non-Muse MSC IV. **C** Representative Laser Doppler blood perfusion images at pre- and 14 days post-operation (POD), and quantitative analysis of the ischemic/non-ischemic limb blood flow ratio over time. P < 0.01 PBS vs. Muse; P < 0.001 PBS vs. Muse; #P < 0.05 non-Muse vs. Muse; ###P < 0.001 non-Muse vs. Muse. The figures were reproduced with permission (Hori et al. 2022). **E** Rat erectile function disorder model that received human BM-Muse cell and non-Muse MSC IV. Panels show representative ICP responses and AP to electrical stimulation of the pelvic nerve in the sham, vehicle, Muse, and non-Muse MSC groups at 28 days. The graph shows ICP/MAP at 28 days. The ICP/MAP in the Muse group was significantly increased compared with that in the vehicle and the non-Muse MSC groups (*P < 0.05), The figures were reproduced with permission (Koyama et al. 2024). **F** Mouse chronic kidney disease model (focal segmental glomerular sclerosis; FSGS) that received human BM-Muse cell and non-Muse MSC IV. Muse cells integrated into the damaged glomerulus, and differentiated into podocyte marker podocin( +), mesangial cell marker megsin ( +), and endothelial cell marker von Willebrand Factor ( +) cells at 7 weeks. On the other hand, only a few non-Muse MSCs integrated into the glomerulus; thus, their differentiation into glomerular cells was not detected. The graph shows that the Muse group showed significantly higher creatinine clearance compared with the vehicle (P < 0.001) and non-Muse (P < 0.01) groups at 5 weeks. Scale bars, 20 µm.The figures were reproduced with permission (Uchida et al. 2017b).

Muse cells were shown to differentiate into Troponin( +)-/actinin( +)-/Connexin43( +)-cardiomyocytes and CD31( +)-vascular cells in vivo (Yamada et al. 2018). Intravenously injected human xenogenic Muse cells engrafted into the rabbit ischemic heart tissue were shown to be differentiated into physiologically functional cardiomyocytes, exhibiting Ca2 + influx and efflux synchronous with systole and diastole. The Muse group showed a statistically significantly reduced infarct size, improved left ventricle (LV) function, and attenuated LV remodeling compared to the MSCs and non-Muse MSC groups at 2 weeks and 2 months (Fig. 7A, B) (Yamada et al. 2018). Muse cells also contributed to neovascularization by differentiating into CD31( +) vascular cells in the ischemic heart tissue (Yamada et al. 2018).

In hindlimb ischemia, aortic dissection, and aortic aneurysm mouse models, intravenously injected human BM-Muse cells were selectively integrated into the damaged vessels and spontaneously differentiated into both CD31(+) endothelial cells and α-smooth muscle actin (+) smooth muscle cells that comprise the vascular system (Hori et al. 2022). Muse cells also produced trophic factors that protect the vessels and muscles: VEGF, angiopoietin 1, PDGF, and IGF. Consequently, the Muse group showed better blood supply, muscle fiber mass preservation, and suppression of interstitial fibrosis in the hindlimb ischemia model (Fig. 7C, D) (Hori et al. 2022).

In mouse aortic aneurysm and aortic dissection models, human Muse cells integrated into the lesion site and differentiated into both endothelial and smooth muscle cells, and the latter successfully produced elastic fibers in the tunica media of the aorta (Hosoyama et al. 2018; Takahashi et al. 2024).

**Clinical trial:** An open-label, non-randomized, single-arm, non-controlled clinical trial was conducted in ST-elevation myocardial infarction patients with LV ejection fraction (LVEF) ≤45% after successful percutaneous coronary intervention (PCI) (Noda et al. 2020). Patients received an intravenous drip of 15 million donor-Muse cells within 5 days after disease onset. LVEF, measured by echocardiography with the modified Simpson’s method, was 40.7 ± 1.5% before administration and increased significantly to 52.0 ± 2.6 % at 12 weeks (p < 0.001). Serious adverse effects did not occur (Noda et al. 2020). The significant increase in the LVEF of more than 10% in this clinical trial is expected to improve the long-term prognosis of AMI.

### Erectile dysfunction

In the rat erectile dysfunction model made by cavernous nerve injury, intravenously injected human BM-Muse cells showed statistically significant recovery in the mean intracavernous pressure (ICP)/arterial pressure (AP) values compared to the human non-Muse MSC treated group at 28 days (Fig. 7E) (Koyama et al. 2024). In the major pelvic ganglion, Muse cells were observed to be engrafted at 48 h and expressed Schwann cell markers S100 and GFAP at 28 days, while non-Muse MSCs were not detected at 48 h (Koyama et al. 2024). Muse cell IV may therefore recover erectile dysfunction after cavernous nerve injury.

### Kidney

Muse cells were shown to differentiate into glomerular cells by phagocytosing apoptotic glomerular cell fragments in vitro (Wakao et al. 2022). In the Adriamycin-induced chronic kidney disease mouse model, intravenously injected human BM-Muse cells selectively homed into the kidney and differentiated into podocin ( +)/WT1( +) podocytes, megsin ( +) mesangial cells, and von Willebrand factor ( +)/CD31( +) endothelial cells in the glomerulus, while only a few human non-Muse MSCs were detected in the kidney after 5 and 7 weeks (Fig. 7F) (Uchida et al. 2017b). The improvements in urine protein levels, creatinine clearance, plasma creatinine levels, and kidney fibrosis suppression was statistically significant in the Muse cell group compared to that of the non-Muse MSC group at 7 weeks (Fig. 7G) (Uchida et al. 2017b).

### Liver

**Fig. 8:**
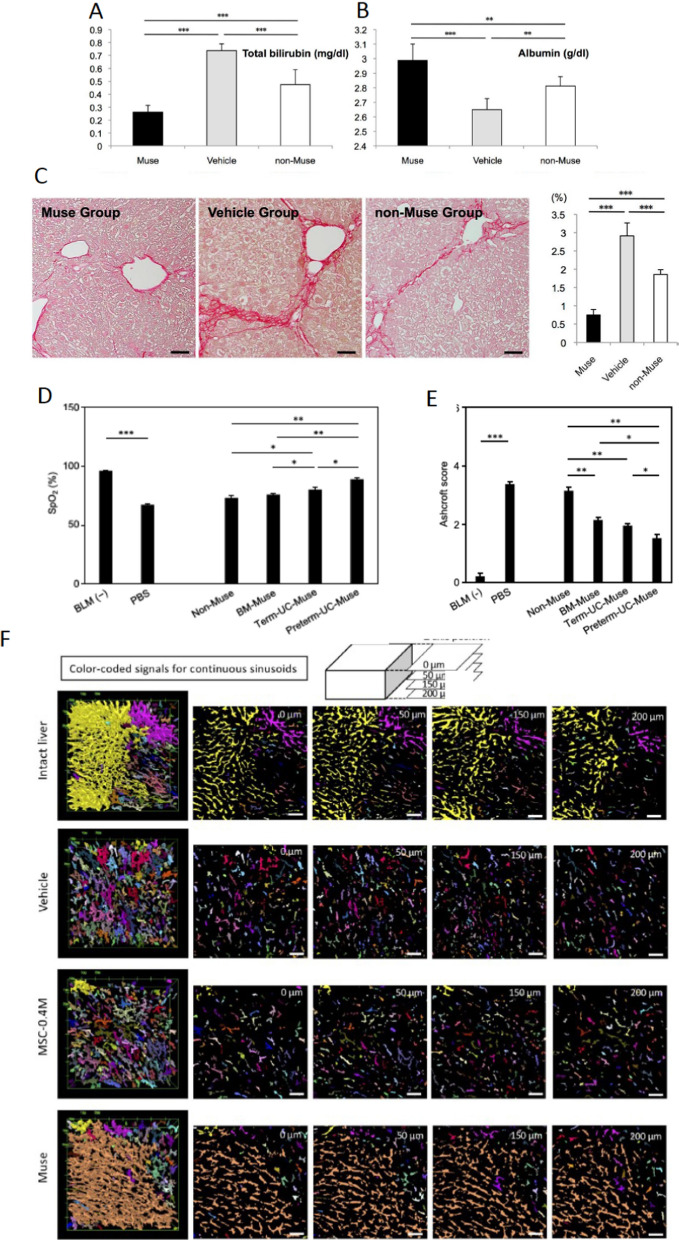
Comparison of therapeutic effects between Muse cells, non-Muse MSCs, and MSCs (Part 3). **A**–**C** Mouse liver cirrhosis (fibrosis) model that received human BM-Muse cell and non-Muse MSC IV. Serum total bilirubin (**A**) and albumin (**B**) levels in the Muse, vehicle, and non-Muse groups at 8 weeks of infusion. **C** The liver fibrotic area was evaluated by staining with **D** Sirius red at 8 weeks. **p < 0.01, ***p < 0.001. Scale bars: 50 μm. The figures were reproduced with permission (Iseki et al. 2017). **D**, **E** Rat bleomycin-induced lung injury model that received human BM-Muse cell, non-Muse MSC, term UC-Muse cell, and preterm UC-Muse cell IV. **D** Pulmonary function measurement, oxygen saturation (SpO2) at day 9. **E** Lung fibrosis assessment. The lung sections stained by hematoxylin–eosin were scored according to the Ashcroft scale. *p < 0.05, **p < 0.01, and ***p < 0.001. The figures were reproduced with permission (Win et al. 2024). **F** Rat extra-small partial liver transplantation model that received human BM-Muse cell and MSC IV. (A) Glasbey-on-dark processing was used to color each continuous sinusoid with a distinct color to clarify the detailed organization of the sinusoids. Continuous sinusoids were more abundant in the liver of the intact and Muse groups, while the majority of sinusoids in the liver grafts were discontinuous in the vehicle and MSC groups. The figures were reproduced with permission (Shono et al. 2021).

Muse cells were shown to differentiate not only into albumin ( +)/HepPar-1( +) hepatocytes but also into Lyve-1( +) sinusoid endothelial cells, cytokeratin 7( +) cholangiocytes, and CD68( +) Kupffer cells in vivo (Iseki et al. 2017, 2021; Katagiri et al. 2016; Wakao et al. 2022). Few MSCs and non-Muse MSCs, however, differentiated into the liver components (Iseki et al. 2017, 2021). Human Muse cells expressed endodermal markers, Prox1, keratin18, and α-fetoprotein at day 3, and albumin at day 14 after phagocytosing apoptotic mouse liver fragments (Wakao et al. 2022). The selective migration into damaged liver, improvement in albumin, total bilirubin, AST, and ALT, and suppression of necrosis and fibrosis were statistically significant in the human and swine BM-Muse cell treated group as opposed to the MSC and non-Muse MSC treated groups in the mouse chronic liver fibrosis and swine hepatectomy models (Fig. 8A, C) (Iseki et al. 2017, 2021).

### Lung

Muse cells have been shown to differentiate into type I alveolar cells positive for aquaporin5 and podoplanin and type II alveolar cells positive for proSP-C and surfactant protein C both in vivo and in vitro (Wakao et al. 2022; Win et al. 2024). In the bleomycin-induced lung injury model, intravenously injected human BM-, term UC-, and preterm-UC-Muse cells homed into the lung at a higher ratio than human BM non-Muse MSCs (Win et al. 2024). The BM- and UC-Muse cell groups showed better recovery in oxygen saturation, Ashcroft and modified ATS scales, and suppression of surfactant protein D serum level compared to the non-Muse MSC group with statistical significance (Fig. 8D, E). Among the three types of Muse cells, the preterm-UC-Muse cell group consistently showed a statistically higher homing rate to the lung and better functional recovery than the term-UC-Muse cell and BM-Muse cell groups (Win et al. 2024).

### Digestive system

Human Muse cells were shown to differentiate into LGR5( +), ATOH1( +), ELF3( +), and FABP2( +) intestinal cells by phagocytosing apoptotic mouse intestinal cells in vitro (Wakao et al. 2022). Human UC- and BM-Muse cell IV rescued lethal radio-induced gastrointestinal syndrome by intestinal regeneration, suppression of apoptosis, and trophic effect (Dushime et al. 2023; Miura et al. 2024).

### Cornea

Human adipose-Muse cells transplanted into mouse and tree shrew wounded corneas suppressed scarring, inflammation, and neovascularization, leading to the increase of corneal re-epithelialization and nerve regrowth by differentiation into corneal stromal cells. Improvement of stimulated visual acuity was confirmed (Guo et al. 2020).

### Tissue protection, anti-inflammation, immunomodulation, and anti-fibrosis

Similar to MSCs, Muse cells produce factors related to anti-inflammation and immunomodulation, such as granulocyte-colony stimulating factor (G-CSF), IL-1ra, IL-10, TGF-β, NO, IDO, Prostaglandin E2; tissue protection and regeneration, such as keratin growth factor, angiopoietin-1, HGF, VEGF, EGF, IGF-1; anti-fibrosis, such as MMP-1, -2, -9. Some factors were highly produced by MSCs and others by Muse cells (Ozuru et al. 2020; Uchida et al. 2017b; Yabuki et al. 2018; Yamada et al. 2018) However, since Muse cells integrated into the damaged tissue, survived, and supplied these factors to the tissue, while on the other hand, MSCs and non-Muse MSCs did not migrate or integrate into the damaged tissue, trophic effects were much more potent in the Muse cell treated group rather than the MSC and non-Muse MSC treated groups (Iseki et al. 2017; Uchida et al. 2017b; Yabuki et al. 2018; Yamada et al. 2018) One example is the effectiveness of human Muse cells and non-Muse MSC IV in Vero toxin-producing E. coli-associated encephalopathy mouse model (Ozuru et al. 2020) Muse cells that reached the brain and spinal cord suppressed inflammation, apoptosis, destruction of blood–brain-barrier, and gliosis, mainly by G-CSF production, while non-Muse MSCs did not reach the brain and spinal cord. Consequently, all of the Muse cell group survived, while the effectiveness of the non-Muse MSC group was substantially lower compared to that of the Muse group (Ozuru et al. 2020).

### Vascular protection effect

Since Muse cells quickly migrate and home into the damaged tissue moreso than MSCs and non-Muse MSCs, even if all of them have a similar ability to produce vascular protection factors such as HGF and VEGF, Muse cells have a higher vascular protection effect than the other two in the acute phase. In a rat extra-small partial liver transplantation model, where liver sinusoid endothelial cells are injured by shear stress after portal reflow, the Muse cell group showed better capillary blood flow and protection of sinusoid endothelial cells than those in the MSCs group at 3 days after IV (Fig. 8F) (Shono et al. 2021).

Muse cells showed a statistically significant therapeutic effect in the above-mentioned organ injury models compared to non-Muse MSCs and MSCs. In addition, the intravenous administration of Muse cells leads to organ repair and functional recovery, suggesting that the intravenous administration of Muse cells may be effective against aging-related organ dysfunctions and degeneration.

## Is there any merit in purifying Muse cells from MSCs?

**Fig. 9:**
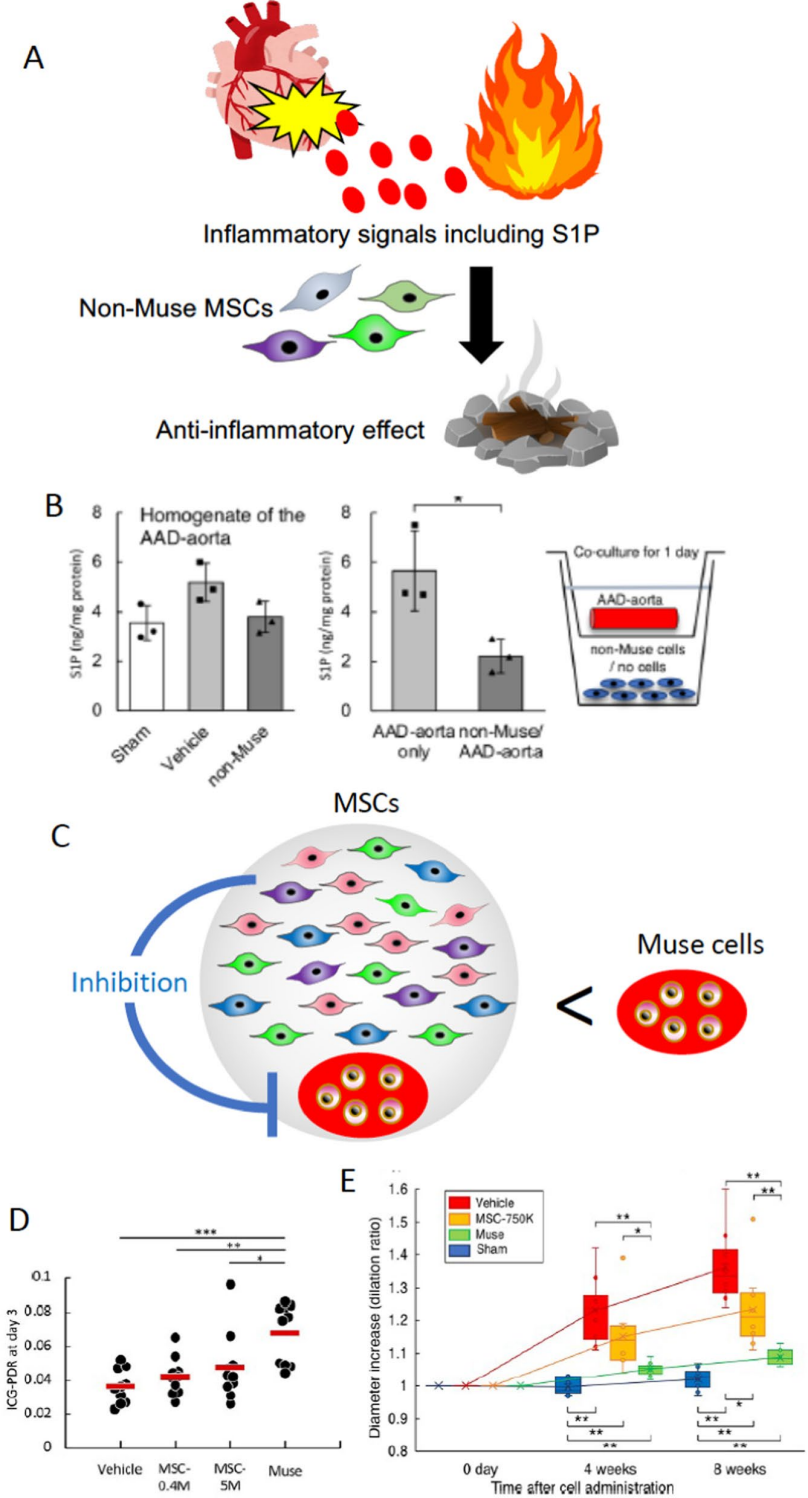
A smaller number of purified Muse cells is highly effective than a large number of MSCs. **A** The schema explains how non-Muse MSCs suppress S1P, the critical signal for Muse cell migration and homing to the damaged site. **B** Mouse aortic dissection model that received human BM-non-Muse MSCs. (Left) S1P level of the dissected aorta homogenate supernatant in each group at 1 day after cell administration. Although without statistical significances, non-Muse MSC treated aorta showed the reduction of the S1P level comparable to the sham level. (Right) Schematic diagram of co-culture using the Boyden chamber. S1P level of the upper chamber, which contains the dissected aorta fragment, was measured at 24 h after co-culture in the acute aortic dissection (AAD)-aorta only group (aorta at 1 day after AAD onset was cocultured with no cells) and non-Muse MSC/AAD-aorta group (aorta at 1 day after AAD onset was cocultured with 712,000 non-Muse MSCs). The S1P level substantially reduced in the non-Muse/AAD-aorta group compared to the AAD-aorta only group. *: p < 0.05. The figures were reproduced with permission (Takahashi et al. 2024). **C** Schematic diagram explaining the experiments in (D) and (E). The MSC group contained the same number of Muse cells as the Muse group. **D** Rat extra-small partial liver transplantation model that received human BM-Muse cell and MSC IV. The ICG-PDR, an indicator of dynamic liver function, was the highest in the Muse group (4 × 10^5^ Muse cells), with statistical significance to the MSC-5 M group (5 million MSCs that contain 4 × 10^5^ Muse cells, the same number of Muse cells as the Muse group)(p < 0.05). The MSC-0.4 M group (4 × 10.^5^ MSCs) received the same number of MSCs as the Muse group. Notably, there was no significance between the MSC-5 M and MSC-0.4 M groups. The figures were reproduced with permission (Shono et al. 2021). **E** Mouse aortic dissection model that received human BM-Muse cell and MSC IV. The dilation ratio of the dissected aorta at 4 and 8 weeks after cell administration. The Muse group showed statistically significant better recovery than the MSC-750 K group that contain the same number of Muse cells as the Muse group at 8 weeks (p < 0.05). The figures were reproduced with permission (Takahashi et al. 2024).

Since Muse cells constitute only a small proportion of an entire MSC pool, a simple question arises: could a large dose of MSCs have the same effect as a small amount of purified Muse cells? The answer is no. This is due to the fact that the action of a small percent of Muse cells is largely inhibited by co-existing non-Muse MSCs that occupy 97–98% of MSCs. Non-Muse MSCs have a strong anti-inflammatory effect. When MSCs were intravenously injected, a great amount of non-Muse MSCs suppressed S1P production in the damaged tissue (Fig. 9A, B). If S1P is suppressed, Muse cells are unable to find the damaged site; thereby, impeding the first step in tissue repair (Takahashi et al. 2024). In this manner, non-Muse MSCs inhibit Muse cell activity within the entire MSC pool (Fig. 9C).

The following experiments were conducted to compare the therapeutic effect of a small number of purified Muse cells and a large number of MSCs that contain the same number of Muse cells (Fig. 9C–E). In the rat extra-small partial liver transplantation model, the Muse group (received 4 × 10^5^ human BM-Muse cells) exhibited statistically superior liver sinusoid protection effect than the MSC group (5 × 10^6^ human BM-MSCs that contain 4 × 10^5^ Muse cells)(Fig. 9D) despite the same number of total Muse cells (Shono et al. 2021). In the acute aortic dissection model, suppression of diameter expansion was greater in the Muse group (received 50,000 human BM-Muse cells) than in the MSC group (received 750,000 human-MSCs that contained 50,000 Muse cells) with statistical significance (Fig. 9E) (Takahashi et al. 2024).

There is no doubt that MSCs themselves possess therapeutic effects. However, compared to a small number of purified Muse cells, Muse cells have more powerful, clear-cut effects on tissue repair and functional recovery than MSCs. In fact, in preclinical studies comparing Muse with MSCs and non-Muse MSCs, the Muse cell group consistently showed statistically significant structural and functional improvements in every organ injury model.

## Future perspectives

A prerequisite for applying stem cells for use in anti-aging therapy is that safety concerns, such as tumorigenesis, must be minimized. In this respect, MSCs and Muse cells are somatic stem cells and do not inherently have tumorigenicity, making them both feasible candidate stem cells.

MSCs have been administered to humans for various diseases in clinical trials and are effective in treating aging to an extent. Based on preclinical and clinical studies on Muse cells, donor Muse cell IV is expected to deliver a superior therapeutic effect than MSC IV. Directly using donor Muse cells without an HLA-matching test and immunosuppressant treatment is of great benefit in anti-aging therapy; allogenic and xenogenic Muse cells administered by intravenous infusion were shown to survive in the recipient tissue without immune rejection for an extended period without an HLA-matching test and immunosuppressant treatments in preclinical studies (Fujita et al. 2021b; Iseki et al. 2021; Suzuki et al. 2021; Yamada et al. 2018).

For use in anti-aging therapy, it would be more rational to use Muse cells from active young tissues rather than autologous ones from elderly people. Although, with a small number of samples, a significant negative correlation between the number of Muse cells per weight and donor age is reported in adipose tissue (r2 = 0.75, p < 0.05; 11 donors aged 4–88 years) (Yamauchi et al. 2017b). In that respect, directly infusing younger active Muse cells, such as UC-Muse cells, is of great benefit in anti-aging therapy, as they can be administered repeatedly without burdening the patient and donors.

Even though the number of Muse cells administered in clinical trials was far smaller than that of MSCs (one-tenth or one-fifteenth), the significant therapeutic effect was confirmed in multiple diseases (Fujita et al. 2021b; Koda et al. 2024; Niizuma et al. 2023; Noda et al. 2020; Sato et al. 2024; Yamashita et al. 2023). Issues for future consideration include (i) determining the best source of Muse cell, (ii) the optimal number of cells to be administered in one dose, and (iii) the desirable interval between administrations.

## Which source of Muse cells is suitable for anti-aging treatment?

Muse cells can be obtained from various sources. As the lung injury model showed, young tissue-derived Muse cells, i.e., UC-Muse cells, were more effective at restoring the structure and function than adult tissue-derived Muse cells, e.g., BM-Muse cells. In UC-Muse cells, those from preterm infants were more effective than those from term infants (Win et al. 2024). From these, younger tissue-Muse cells are considered more effective for anti-aging therapy.

However, when comparing UC- and BM-Muse cells, one weakness of UC-Muse cells has been pointed out: BM-Muse cells have a higher ability to differentiate into vascular cells than UC-Muse cells (Win et al. 2024). Therefore, when the treatment target is vascular regeneration, using UC-Muse cells might be a more logical strategy than using BM-Muse cells. As BM-Muse cells have been shown to have a statistically significant therapeutic effect in vascular diseases in preclinical studies, such as hind limb ischemia, aortic aneurysm, and aortic dissection models, compared to MSCs and non-Muse MSCs, comparison of UC-Muse and BM-Muse cells in multiple vascular system disease models remains a topic for future study (Hori et al. 2022; Hosoyama et al. 2018; Takahashi et al. 2024).

## Single-dose vs. multiple-dose Muse cells

There was no significant difference in therapeutic outcomes in acute injuries between single and multiple Muse cell administrations in the preclinical study (Hosoyama et al. 2018). Still, multiple administrations over a specific period may be more effective for chronically progressing diseases (Yamashita et al. 2023).

In the acute aortic aneurysm mouse model, a single-dose human BM-Muse cell IV, and three doses (at day 0, 7, and 14) of Muse cells IV did not show significant differences in the reduction of aortic dilation and repair of the aortic tissue (Hosoyama et al. 2018). On the other hand, in the clinical trial for ALS that received donor-Muse cell IV every month for six months, the progression of the score was efficiently inhibited (Yamashita et al. 2023). Since aging is a phenomenon that progresses slowly and continuously, the treatment should not be a one-off but rather multiple therapeutic interventions spaced over a specific period of time.

## Other possibilities in Muse cells

Senolytic drugs selectively induce the death of senescent cells. Since Muse cells can differentiate into the same cell type as the cells they phagocytose and replace the damaged cells with healthy functional cells, combining senolytics and Muse cells might be one of the possible approaches. Combining Muse cell treatment with other anti-aging approaches, such as cosmetic treatment, platelet-rich plasma therapy, and EVs, is also expected to deliver synergistic effects.

The relationship between aging and immunological activity is another topic in anti-aging since the immune system undergoes a gradual decline in function with aging, a process known as immunosenescence (Guarente et al. 2024). How Muse cells can affect immunosenescence remains to be determined in the future.

## Data Availability

No datasets were generated or analysed during the current study.

## Abbreviations

AP: Arterial pressure
BDNF: Brain-derived neurotrophic factor
bFGF: Basic fibroblast growth factor
BM: Bone marrow
EGF: Epidermal growth factor
ES: Embryonic stem
EVs: Extracellular vesicles
G-CSF: Granulocyte-colony stimulating factor
HGF: Hepatocyte growth factor
ICP: Intracavernous pressure
IDO: Indoleamine 2,3-dioxygenase
IGF: Insulin-like growth factor
IL: Interleukin
iPS: Induced pluripotent stem
KGF: Keratinocyte growth factor
LV: Left ventricle
LVEF: LV ejection fraction
mRS: Modified Rankin scale
Muse: Multilineage-differentiating stress enduring
MSC: Mesenchymal stem cell
NGF: Nerve growth factor
PDGF: Platelet-derived growth factor
PGE2: Prostaglandin E2
SASP: Senescence-associated pro-inflammatory secretory phenotype
SSEA: Stage-specific embryonic antigen
S1P: Sphingosine-1-P
TGF-β: Transforming growth factor-beta
TLR2: Toll-like receptor 2
TSG-6: Tumor necrosis factor-stimulated gene-6
UC: Umbilical cord
VEGF: Vascular endothelial growth factor
